# Social factors in frequent callers: a description of isolation, poverty and quality of life in those calling emergency medical services frequently

**DOI:** 10.1186/s12889-019-6964-1

**Published:** 2019-06-03

**Authors:** Gina Agarwal, Janice Lee, Brent McLeod, Sabnam Mahmuda, Michelle Howard, Krista Cockrell, Ricardo Angeles

**Affiliations:** 1 Department of Family Medicine, McMaster University, David Braley Health Sciences Centre, 100 Main Street West, 6th Floor, Hamilton, ON L8P 1H6 Canada; 2 McMaster University, 100 Main Street West, 6th Floor, Hamilton, ON L8P 1H6 Canada; 3 Hamilton Paramedic Services, Hamilton, Canada; 4 SickKids Centre for Global Child Health, 100 Main Street West, 6th Floor, Hamilton, ON L8P 1H6 Canada; 5 Department of Family Medicine, McMaster University, 100 Main Street West, 6th Floor, Hamilton, ON L8P 1H6 Canada; 6 Western Sydney University, School of Science and Health, Hamilton, Canada; 7 University of Western Sydney, Locked Bag 1797, Penrith, NSW 2751 Australia

**Keywords:** Health services, Emergency medical services, Frequent callers, Social factors, Poverty, Quality of life, Social isolation

## Abstract

**Background:**

Frequent users of emergency medical services (EMS) comprise a disproportionate percentage of emergency department (ED) visits. EDs are becoming increasingly overwhelmed and a portion of use by frequent callers of EMS is potentially avoidable. Social factors contribute to frequent use however few studies have examined their prevalence. This study aims to describe social isolation/loneliness, poverty, and quality of life in a sample of frequent callers of EMS in the Hamilton region, a southern Ontario mid-sized Canadian city.

**Study design:**

Cross-sectional quantitative study.

**Methods:**

We surveyed people who called EMS five or more times within 12 months. A mailed self-administered survey with validated tools, and focused on four major measures: demographic information, social isolation, poverty, and quality of life.

**Results:**

Sixty-seven frequent EMS callers revealed that 37–49% were lonely, 14% had gone hungry in the preceding month, and 43% had difficulties making ends meet at the end of the month. For quality of life, 78% had mobility problems, 55% had difficulty with self-care, 78% had difficulty with usual activities, 87% experienced pain/discomfort, and 67% had anxiety/depression. Overall quality adjusted life years value was 0.53 on a scale of 0 to 1. The response rate was 41.1%.

**Conclusions:**

Loneliness in our participants was more common than Hamilton and Canadian rates. Frequent EMS callers had higher rates of poverty and food insecurity than average Ontario citizens, which may also act as a barrier to accessing preventative health services. Lower quality of life may indicate chronic illness, and users who cannot access ambulatory care services consistently may call EMS more frequently. Frequent callers of EMS had high rates of social loneliness and poverty, and low quality of life, indicating a need for health service optimization for this vulnerable population.

## Background

In recent decades, emergency medical service (EMS) use has increased dramatically, straining emergency departments (ED) beyond their capacity and representing a significant cost in the healthcare budget. [1] Between 2012 and 2014 alone, Ontario ambulance use has increased by 8%, representing an increase of 100,000 dispatches and 17% in costs. Out of all calls, 58,000 patients (58%) are transported and 48,000 (48%) are not. [2] Some emergency service use among frequent callers is likely preventable, and may represent a discrepancy between physician and patient perceptions of medical emergencies. [3–7] In previous literature surveying emergency service use, one out of three ambulance dispatches have not been perceived as medical emergencies by health services researchers, [8] and frequent callers account for up to 40% of transports. [9–14] In specifically Canadian studies, frequent callers comprise 2.1–3.6% of overall ED users but account for 9.9–13.8% of visits. Frequent callers have been defined as people who call 4 to 5 or more times within 1 year, [15, 16] though definitions range from 3 to 10 times per year. [16–20]

Existing literature from the United States of America (USA) characterizing frequent ED and EMS users (as opposed to callers) reveals that they are often vulnerable populations [15, 21] who tend to be of lower socioeconomic status, [22] have psychiatric and substance use disorders, [23] or have chronic medical conditions (often with multiple comorbidities). [4, 24–28] Common chronic condition exacerbations were found to be in ambulatory care sensitive diseases such as asthma, [29, 30] chronic obstructive pulmonary disease, renal failure, and sickle cell anemia. [23] Consistent with this, frequent EMS users have been found to be high users of ambulatory care services (outpatient medical care that prevents or reduces hospitalizations). [31] Amongst non-specific presenting complaints, nausea and vomiting, chest pain, anxiety, pain, and shortness of breath are most common, which are not necessarily differing from non-frequent callers. [9, 31] Frequent EMS users also have poorer self-rated general health, [32] higher mortality rates post-ED, [33] hospital admissions, [34] and higher rates of ambulance usage. [34]

A growing body of literature studies potential psychosocial factors behind frequent ED usage. Some propose that frequent EMS users lack proper access to primary healthcare services and are forced to rely on emergency health services as their only source of regular medical care, thus presenting for non-urgent health issues. [35–37] Ambulatory care-sensitive medical conditions such as asthma, diabetes, chronic obstructive pulmonary disease and congestive heart failure are one example where patients rely heavily on close monitoring in outpatient health services; without this, they are more likely than others to require ED visits and unscheduled hospitalization. [37] Social isolation and loneliness have also been identified as predictors of frequent ED usage (lacking close friends, living alone, unemployment, disability retirement, and subjective feelings of loneliness). [1, 30, 33, 38–40] These patients may also have emotional, cognitive, and stress-related neuroendocrine, cardiovascular and immune changes that contribute to difficulty managing their health. [40, 41] The increasing proportion of elderly citizens who live alone is another potential reason for recent increases in ED visits amongst the elderly. [42] Lastly, frequent EMS users have higher rates of poverty, which is associated with a higher prevalence of chronic illnesses, as well as barriers to preventative and primary healthcare services. [43, 44] Additionally, increasing ED use has been associated with homelessness and unstable housing status, further emphasizing the vulnerability of this population. [21] However, the extent to which social factors actually determine ED and EMS usage has not yet been determined.

Existing literature has largely focused on the characteristics of frequent users of EDs, rather than callers to EMS, who are a different population. [5] Most research regarding frequent users has taken place in large American cities, and has used differing definitions of a frequent user, and tended to focus on psychiatric illness, failing to describe their actual characteristics. There are far fewer primary studies in mid-sized cities and in Canada, which have vastly different health service infrastructure than USA (i.e. universal health insurance). Given that a large proportion of frequent users of ED services arrive by ambulance (59.3% of frequent users vs. 12% of the general population), [5, 15, 17, 45–49] studying this population of frequent callers to EMS will aid in continuing to find a solution to ED overcrowding. Investigating the profile of social factors will assist in planning primary and preventative health services development in these subpopulations of frequent EMS callers.

This study aims to describe social isolation/loneliness, poverty, and quality of life in a sample of frequent users of EMS in the Hamilton region, a southern Ontario mid-sized Canadian city.

## Methods

### Study design and sample

This cross-sectional quantitative study surveyed participants who had called 911 at least five times between April 1st 2015 and March 31, 2016. Participants were residents of the City of Hamilton 18 years or older, and were invited to participate by the Hamilton Paramedic Service, who had extracted their names from their database. The survey was distributed in two occurrences for ease of workload; initially commencing May 2016 and then a second sample commencing September 2017 each time using a modified Dillman’s Total Design. The surveys were mailed with an introductory letter with study objectives and explanation, instructions for return, a pre-stamped envelope, and a $5 gift card. A second mail-out included a reminder letter and was sent 1 week after initial mail-out to non-responding participants. A final mail-out 3–7 weeks later included a replacement introductory letter, instructions for returning the survey, the survey itself, and a pre-stamped envelope. When participants returned the survey, they were given an additional $5 gift card.

### Measures

The survey questions focused on the following: (1) demographic information, (2) social isolation, (3) poverty, and (4) quality of life.

Demographic information collected included age, sex, body mass index (BMI) and employment status. To measure social isolation and loneliness, two well-validated scales were used since they measured different aspects of loneliness; the UCLA 3 Item Loneliness scale which could quantify loneliness, [50] and a portion of the De Jong Gierveld 6-Item Loneliness Scale which measured social loneliness. [51] To measure poverty, two highly sensitive and specific clinical screening questions were used – whether participants had trouble making ends meet at the end of the month, [52] and whether either they or their family members had gone hungry in the past month. [53] To measure quality of life, we used the EQ5D-3 L, a 5-item preference based instrument for 5 health states at 3 levels (mobility, self care, usual activities, pain/discomfort, anxiety/depression). [54] The scores were converted according to a Canadian preferences valuation to a score for quality adjusted life years (QALY). [54]

### Ethical considerations

In order to adhere to high ethical standards, the survey was completely separate from any healthcare provided so that participants would not feel pressured or coerced to participate. Therefore, a group that already had difficulty accessing healthcare would not find this to be an additional barrier. The surveys were kept confidential and anonymous. Additionally compensation was provided for the time taken in filling out the survey. These ethical considerations passed Local Research Ethics Board Standards.

### Data analysis

Descriptive statistics were calculated to describe social factors (poverty, loneliness, and quality of life). The mean, median, mode and 25th and 75th quartiles were calculated for QALY. Data was analyzed using SPSS statistical software, version 24. Missing data were excluded from the analyses and the final “valid percent” was taken.

## Results

### Demographics (Fig. 1)

**Fig. 1:**
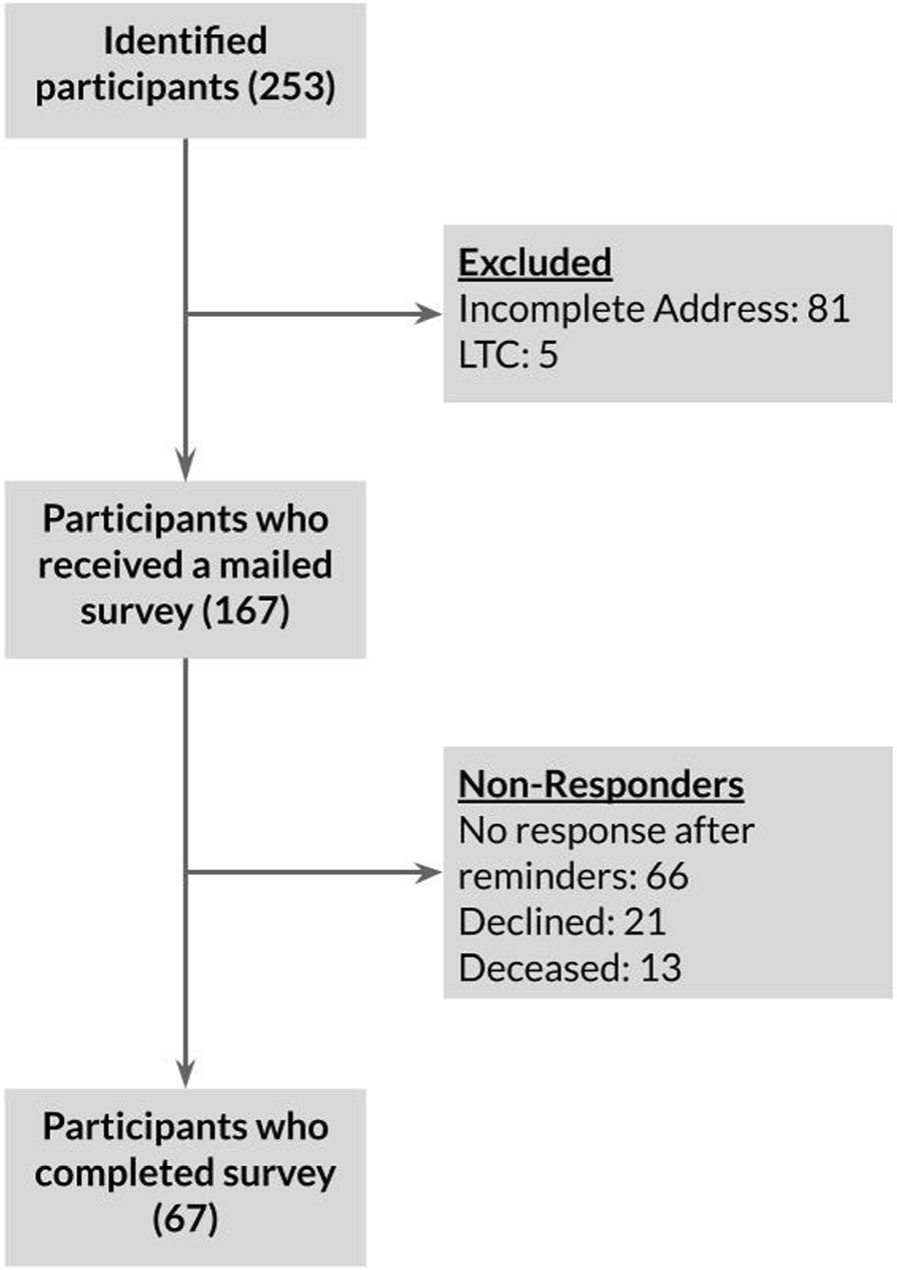
Flow diagram for participants

253 eligible participants were identified. Of those, 81 were excluded as they had given an incomplete address and 5 were excluded as they were from a long-term care facility. 167 people in total were sent a mailed survey, and 67 completed the survey, yielding a response rate of 41.1%. Of the non-responders, 13 were found to be deceased, 21 declined the survey, and 66 did not respond even after reminders (Fig. 1).

Most of the respondents were older than 40 years of age (88.1%) (Table 1). With respect to employment status, 85.1% of the sample was unemployed. For BMI, 19.4% of participants had a normal BMI from 18.5–24.9, while 58.3% of the population was underweight, overweight or obese. Most individuals lived with someone (58.2%) but 38.8% of individuals lived alone.

**Table 1:** Demographic Data

Variable		Frequency (*N* = 67)	%
Age	18–24 yrs	1	1.5
	25–40 yrs	7	10.4
	41–64 yrs	27	40.3
	65–74 yrs	15	22.4
	75+ yrs	17	25.4
Sex	Male	33	49.3
	Female	34	50.7
Employment Status	Employed	9	13.4
	Not employed	57	85.1
	Declined to answer	1	1.5
BMI	Underweight (< 18.5)	3	4.5
	Normal Weight (18.5–24.9)	13	19.4
	Overweight (25.0–29.9)	17	25.4
	Obese Class I (30.0–34.9)	10	14.9
	Obese Class II (35.0–39.9)	6	9
	Obese Class III (> = 40.0)	3	4.5
	Declined to answer	15	22.4
Living Arrangement	Lives by self	26	38.8
	Live with one or more family members	32	47.8
	Live with non-family member	7	10.4
	Declined to answer	2	3

Non-responders had a similar age and sex distribution to responders. In terms of sex, 43.3% were female and 56.7% were male. In terms of age, the most represented categories were ages 41–64 (39.4%) and 74+ (31.7%), which are the same categories that were most represented among responders.

### Social factors

On the UCLA 3 Item scale, 49.25% of participants achieved a “lonely” score of 6 to 9, while 47.76% of participants achieved a “not lonely” score of 3 to 5; 20.9% of participants felt that they lacked companionship often, and 40.3% felt that they lacked companionship some of the time. On the De Jong Social Loneliness questions, 37.3% were intensely lonely (Tables 2 and 3).

**Table 2:** UCLA Social Loneliness Score

Score summaries		Frequency	Percent
Lonely (Score = 6–9)		33	49.25
Not Lonely (Score = 3–5)		32	47.76
Declined to answer		2	2.99
Questionnaire responses:	Variables (score)	Frequency	Percent
There are enough people I feel close to	Yes, enough people to feel close to (1)	31	46.3
	More or less people to feel close to (2)	21	31.3
	No people to feel close to (0)	12	17.9
	Declined to answer	3	4.5
How often feel left out	Hardly ever feel left out (0)	27	40.3
	Some of the time feel left out (1)	27	40.3
	Often feel left out (2)	11	16.4
	Declined to answer	2	3
How often feel isolated	Hardly ever feel isolated (0)	27	40.3
	Some of the time feel isolated (1)	24	35.8
	Often feel isolated (2)	14	20.9
	Declined to answer	2	3

**Table 3:** De Jong Social Loneliness Score

Score summaries:		Frequency	Percentage
0 (Not socially lonely)		19	28.4
1		7	10.4
2		12	17.9
3 (Intensely Socially lonely)		25	37.3
Declined to Answer		4	6
Questionnaire responses:	Variables (score)	Frequency	Percentage
Living Arrangement	Lives by self (0)	26	38.8
	Live with one or more family members (1)	32	47.8
	Live with non-family member (2)	7	10.4
	Declined to answer	2	3
People to rely on	Yes there are people to rely on (1)	29	35.8
	More or less, there are people to rely on (2)	24	35.8
	No people to rely on (0)	13	19.4
	Declined to answer	1	1.5
People to trust completely	Many people I can trust completely (yes −1)	27	40.3
	More or less, people can trust completely (2)	18	26.9
	No people I can trust completely (0)	20	29.9
	Declined to answer	2	3
How often do you feel you lack companionship?	Lack companionship hardly ever (0)	26	38.8
	Lack companionship some of the time (1)	27	40.3
	Lack companionship often (2)	14	20.9
	Declined to answer	0	0
Living Arrangement	Lives by self (0)	26	38.8
	Live with one or more family members (1)	32	47.8

Nearly half of respondents (43.3%) reported not having enough money to make ends meet and 14.9% reported that they or a family member went hungry in the past month (Table 4).

**Table 4:** Poverty

Variable		*N*	%
In the past month, was there any day when you or anyone in your family went hungry because you did not have enough money for food?	Yes	10	14.9
	No	55	82.1
	Declined to Answer	2	3
Do you ever have trouble making ends meet at the end of the month?	Yes	29	43.3
	No	37	55.2
	Declined to Answer	1	1.5

### Quality of life

A large percentage (74.6%) of participants experienced some problems walking; 11.9% were completely unable to wash and dress themselves; most had some problems performing usual activities (56.7%); most experienced pain and discomfort and a high proportion (67.1%) experienced moderate or extreme anxiety and depression (Table 5). When the EQ5D-3 L data were converted to QALYs, the mean was found to be 0.533 (out of a range of 0–1, where 1 described perfect quality of life). The 25th and 75th percentiles were 0.376 and 0.664 respectively.

**Table 5:** Quality of Life

Variable		*N*	%
Mobility	No problems walking	14	20.9
	Some problems walking	50	74.6
	Confined to bed	2	3
	Declined to answer	1	1.5
Self care	No problems	30	44.8
	Some problems (washing and dressing)	29	43.3
	Unable to wash or dress self	8	11.9
	Declined to answer	0	0
Usual activities	No problems performing usual activities	14	20.9
	Some problems performing usual activities	38	56.7
	Unable to perform usual activities	14	20.9
	Declined to answer	1	1.5
Pain/Discomfort	None	8	11.9
	Moderate	34	50.7
	Extreme	24	35.8
	Declined to answer	1	1.5
Anxiety/Depression	None	18	26.9
	Moderate	35	52.2
	Extreme	10	14.9
	Declined to answer	4	6
Quality adjusted life years (QALY)			
Mean	0.53		
Median	0.56		
Mode	0.59		

## Discussion

We conducted a survey of 67 frequent users of EMS in a mid-sized Canadian city and found substantial social isolation, loneliness, income and food insecurity, as well as low quality of life. In the current body of literature, few studies of frequent users measure social isolation/loneliness and quality of life, and most of them survey ED users rather than EMS frequent users. [30, 39, 40] Therefore, our study represents a unique approach to emergency health service usage.

In this study, 37.3 to 49.3% of participants experienced significant degrees of loneliness. Comparatively, Canadian research from 2009 has cited that approximately 19–24% of Canadian seniors lack companionship or wish to participate in more social activities. [55] In Hamilton, in 2006, 15% of senior citizens were estimated to be isolated. [56] The high rates of loneliness found in our study are consistent with existing literature on frequent users of ED. People who live alone, lack friends, are divorced, or lack other social support have been shown to be more likely to be frequent users of ED. [17, 38, 57, 58] Accordingly, 38.8% of our participants live by themselves, a widely used indicator for social isolation, [59] compared to 28.2% Canadians (2016). [60] With respect to potential mechanisms, loneliness and frequent ED use have each been independently linked to increased morbidity, in which chronic illness, poor health behaviours, and poor mental health may result in increased mortality. [25, 39, 41, 61–65] However, studies which show higher rates of loneliness in populations of frequent ED users have not found that rates of chronic illness differ between lonely and non-lonely individuals. [25, 39, 61]

Next, our results indicate that frequent callers to EMS have higher rates of poverty and food insecurity than average Ontario citizen, even those described in our population who are reachable and respond to survey; 14.9% of frequent callers were food insecure, compared to 8.2% of Ontario citizens in 2011. [66] Even more significantly, poverty rates were 43.3% in frequent callers, and 8.8% in Ontarians in 2014. [67] Frequent ED use has previously been associated with poverty in USA studies, where lack of medical insurance was a factor in the delay of seeking other primary and preventative healthcare. [3, 35] The presence of higher rates of poverty in our population is significant, as it is likely to suggest that factors other than lack of medical insurance contribute to frequent ED use behaviours. Besides insurance coverage, poverty can still represent a barrier to primary and preventative health services access in the form of lacking transportation to appointments, not being able to take time off work for appointments, or lack of money to pay for prescription drugs. [68]

Thirdly, participants in our study experienced a lower quality of life than Canadian population averages. In each of the 5 dimensions measured in the EQ5D-3 L, a significantly higher percentage experienced some or extreme problems: mobility (77.6% vs 22%), self care (55.2% vs. 4%), usual activities (77.6% vs. 23%), pain/discomfort (86.5% vs. 51%), and anxiety/depression (67.1% vs. 31%). [54] The most significant differences include difficulties in usual activities (54%), mobility (56%) and self care (51%), which may represent the most significant contributions toward EMS calls in frequent users. Previous studies have found high rates of mobility problems and functional decline in frequent users of ED, and that difficulty in activities of daily living are contributory to the decision to present to ED. [69, 70] Additionally, high rates of ambulatory care conditions have been reported in frequent users, the most common being pain-related conditions. [31, 37] This is consistent with the high prevalence of pain and discomfort found in our study.

Lastly, demographics of frequent EMS callers in our study are largely consistent with existing literature, which primarily studies frequent ED users. The majority of frequent users are younger than 65 years, [9, 61] and an equal number of males and females are frequent users of ED. [4, 14, 27, 71, 72] Other studies have described that younger users are more likely to be ED “superusers” (those with 15 or more annual ED visits), though unfortunately no studies have been conducted on similar statistics for EMS callers. [4, 9, 61] Our population’s unemployment rate was 85% – however, a limiting factor may be age, as 47.8% of participants were age 65+. After removing those participants, 37.3% of the population were unemployed, much higher than Ontario’s rate of 5.5%, suggesting unemployment to be a potential contributory factor toward frequent ED usage. [73] Unemployment could also contribute to and result from poverty and social isolation.

The combination of these numerous social factors represents a complex and multifactorial problem that may be an issue unable to be addressed by a purely biomedical approach traditionally used by emergency health services. We propose that a salutogenic approach to health service provision may be beneficial. Unlike traditional curative approaches, a salutogenic approach focuses on social factors which have been identified to create wellness. [74] Health is not viewed as being a dichotomous state of the presence or absence of disease, but rather is conceptualised along a health continuum between total health and death. [75] Salutogenic approaches to health are aware that total health may not be achieved in all instances, such as those with chronic illness, however, the overall wellness of the individual can be *improved* through addressing social factors related to the individual need, by linking the person to the appropriate resources for their situation. [76] Because paramedics have unique access to patients’ living environments, a salutogenic approach may be and has shown to be a promising option for paramedic and health service provision to effectively assess and address such social factors in patient populations. [77, 78]

Limitations of this study include the lack of a comparator group of non-frequent callers of EMS. However, given the difficulties in recruiting our population of frequent callers, it may also be difficult to recruit similar non-frequent callers of EMS. Another limitation is that participant recruitment was limited to the Hamilton region. However, this study is applicable to other mid-sized cities in both Canada and USA, and provides insight into healthcare systems with universal coverage, a gap in existing literature. Lastly, although the response rate was 41.1% which could be viewed as low, this rate is quite good for a mail-in survey, and at least the age and sex profile of non-responders matched our sample. [79] Additionally, 13 of the non-responders were found after to be deceased, and given the high mortality rate in this population, additional non-responders may have passed away without notifying the research team. However, this may mean that participants with the most significant and multiple comorbidities may not be represented in our study, as they are most likely to have been deceased.

## Conclusion

Overall, our study describes high rates of social isolation and poverty, and a low quality of life in frequent callers of EMS compared to Canadian and USA averages, and that subpopulations within the frequent users group of EMS callers were largely similar to those in the frequent ED users group already characterized. Our results are consistent with many studies already conducted in USA, UK, Australia, and China, in both urban and suburban EDs. Due to Canada’s unique health service infrastructure, this study proposes a salutogenic approach to health service provision that is directly applicable to Southern Ontario and other mid-sized Canadian and American cities. Future research may be able to further characterize EMS frequent users, and trial preventative programs as well as social support programs by social workers in order to gain additional insight into interventions that may affect social loneliness, poverty and quality of life in frequent users of EMS and ED. [18, 80]

## Abbreviations

BMI: Body mass index
ED: Emergency department
EMS: Emergency Medical Services
QALY: Quality adjusted life years
USA: United States of America
